# Association between Changes in Depressive State and Cognitive Function

**DOI:** 10.3390/ijerph16244944

**Published:** 2019-12-06

**Authors:** Jae Han Kim, Youngjoo Kim, Junhyun Kwon, Eun-Cheol Park

**Affiliations:** 1 Premedical Courses, Yonsei University College of Medicine, Seoul 03722, Korea; jaehan0605@yonsei.ac.kr (J.H.K.); sarahkimskim15@gmail.com (Y.K.); 2 Department of Public Health, Graduate School, Yonsei University, Seoul 03722, Korea; judekwon@yuhs.ac; 3 Institute of Health Services Research, Yonsei University, Seoul 03722, Korea; 4 Department of Preventive Medicine, Yonsei University College of Medicine, Seoul 03722, Korea

**Keywords:** depression change, cognitive function, cognitive impairment

## Abstract

Dementia is defined as a severe form of cognitive impairment. Research concerning the two-way relationship between depression and cognitive impairment has been conducted; however, there has been little analysis of cognitive function following changes in depressive status. This study describes the association between changes in depressive state and cognitive function in a Korean geriatric population sample. Using the Korean Longitudinal Study of Aging (KLoSA) database, Mini-Mental State Examination (MMSE) scores and Center for Epidemiologic Studies Depression Scale (CESD-10) indexes were used for measuring cognitive function and depression, respectively. The survey population was divided into four case categories by change in depressive status: normal to normal (Group A), normal to depressive (Group B), depressive to normal (Group C), and depressive to depressive (Group D). Analysis of variance, multiple regression analysis, and subgroup analysis were used for statistical examination. In the multiple regression analysis between MMSE values and depressive status change groups, with Group A as the reference, β in all other groups was negative, and its absolute value was large in the order of D, B, and C in both men (B: −0.717, C: −0.416, D: −1.539) and women (B: −0.629, C: −0.430, D: −1.143). There were also significant results in the subgroup analysis in terms of age, working status, participation in social activities, regular physical activities, and number of chronic medical conditions. In conclusion, both cases—those suffering from depression and those having suffered from it before—experience cognitive impairment. The degree of cognitive function being impaired is greater in the case of depression-onset than that of depression-remission. Age, stimulating activities, and chronic conditions are also strongly relevant to cognitive decline accompanied by changes in depressive state.

## 1. Introduction

One of the many visible trends in an aging population is a decline in cognitive abilities. While such symptoms may be expected as one ages, it may also point to the onset of geriatric neurodegenerative diseases. In particular, dementia is a severe form of cognitive impairment. As one of the most common diseases in the world, the number of patients is expected to increase from around 47 million globally in 2015 to 66 million by 2030 and 115 million by 2050 [1]. Because dementia is accompanied by serious cognitive impairment, affecting one’s everyday life, it is a heavy burden for those with the disease, their families, and society [2].

Among many neuropsychiatric symptoms, depression is reported to be correlated with both cognitive impairment [3,4,5] and dementia [6,7]. In one umbrella review, late-life depression was considered to be a convincing environmental risk factor for all types of dementia, and it is postulated that “depression may be an early reaction to perceived cognitive decline” [8]. In the DSM-5 (Diagnostic and Statistical Manual of Mental Disorders, Fifth Edition), one of the diagnosis criteria for major depressive disorder—“diminished ability to think or concentrate, or indecisiveness, nearly every day”—seems to infer the association between a depressive state and cognitive decline as well [9].

With growing evidence suggesting the possible linkage between depressive symptoms and cognitive decline, numerous researches have been conducted to gain advanced understanding of the association. This research has suggested the negative impact of both depression-onset and depression-remission on cognitive function [4,5,10], which is supported by some studies suggesting that cognitive deficits are at least a persistent feature, if not a core symptom, of depression [4,11,12,13]. However, as these studies only account for cognitive abilities corresponding to a one-sided change in depressive state—‘good to bad’ or ‘bad to good’—direct comparison between changes in cognitive function corresponding to the two opposite depression change directions seems to be inappropriate due to differences in the data employed or variations in the study design. Accordingly, we multi-directionally investigated the association between the change in depressive state and cognitive function, measured by the Center for Epidemiologic Studies Depression Scale (CESD-10) index and Mini-Mental State Examination (MMSE), respectively, using the Korean Longitudinal Study of Aging (KLoSA) database.

## 2. Method

### 2.1. Study Population

The Korean Longitudinal Study of Aging database compiles data from the Korean population aged above 45 years (with the exception of those inhabiting Jeju Island). Every two years, beginning in 2006, a self-reported baseline survey has been conducted of the study population. Every two years since 2007, a self-reported in-depth survey of the study population on topics not examined in the baseline survey has been conducted. Baseline data are categorized into eight groups: population, family (children and grandchildren), family (parents and siblings), health status, employment, income, assets, and subjective quality of life. In this study, however, the first baseline survey was excluded, because CESD-10, one of our key variables, was not surveyed in 2006. Moreover, the most recent survey, the seventh baseline survey, was conducted in 2018, but the data are not yet available for general use. Therefore, in our study we employed baseline survey data from 2008 to 2016, resulting in a total of five datasets.

Baseline characteristics were analyzed for the study population in 2008. Deletion of data with missing values for the key variables as well as covariates resulted in a total of 3031 for the male population and 3958 for the female population. For statistical analysis, each change in depression status in the population from 2008 to 2016, rather than the population number itself, was treated as an individual case.

### 2.2. Variables

For measurement of cognitive function, the Mini-Mental State Examination score was used [14,15]. This was categorized by the KLoSA database as follows: normal (24 or more), mild cognitive impairment (18–23), and dementia (17 or less). We used the mean MMSE score, however, which means that the MMSE score was employed as a raw index, because the MMSE alone is not appropriate for diagnostic purpose [16,17] and we wanted to examine the results in detail.

For measurement of depressive state, the Center for Epidemiologic Studies Depression Scale score was used [18,19]. While the KLoSA database provides the score as a raw index ranging from 0 to 10, we categorized those who score 3 or less as normal and those who score 4 or more as depressive [19] for the purpose of our study. The development of depression in individuals across each survey period, meaning changes in depressive status from the participant’s previous responses, was considered to be a separate case. Thus, if a participant had *n* changes in depression state over the five waves of the survey, he or she would account for *n* cases. Each case was then categorized in order to divide the survey population into four case groups. For simplicity, each group (termed ‘depression change groups’) was assigned a label:

- Group A, normal to normal (without depression for the past two years);
- Group B, normal to depressive (having developed depression in the past two years);
- Group C, depressive to normal (having been cured of depression in the past two years);
- Group D, depressive to depressive (having depression for the past two years).

This categorization allowed the comparison of MMSE scores between depression change groups.

### 2.3. Covariates

Demographic characteristics were included as covariates. The covariates are listed as follows: age (categorical: 45–54, 55–64, 65–74, or 75 years and over), educational level (categorical: elementary school or less, middle school, high school, or university or beyond), region (categorical: metropolitan or rural), working status (categorical: working or non-working), household income (categorical: low, mid-low, mid-high, or high), participation in social activities (categorical: no or yes), perceived health status (categorical: healthy, average, or unhealthy), regular physical activities (categorical: yes or no), smoking (categorical: current, former, or never), alcohol intake (categorical: yes or no), number of chronic medical conditions (categorical: none, 1, or ≥2), weight change of ≥5 kg in the past year (categorical: no change, increase, decrease, or fluctuations), and number of cohabiting generations (categorical: couple, two generations, or over two generations). All multivariable models controlled for all covariates unless stated otherwise.

### 2.4. Statistical Analysis

All analysis was carried out separately for men and women. Analysis of variance (ANOVA) was carried out to obtain descriptive statistics. To confirm the patterns of difference among the interesting variable groups (changes in depressive state), we performed post hoc analysis using the Tukey method. A generalized estimating equation (GEE) model was employed for regression analysis between MMSE scores and the dependent variables, including depression development. Regression coefficients, indicated as β, and standard errors were thus acquired.

Subgroup analysis was performed for in-depth study into interactions between depression and other variables with regards to MMSE scores. Other variables included working status, participation in social activities, regular physical activities, number of chronic medical conditions, and weight change of ≥5 kg in the past year.

All *p*-values were accepted as significant if lower than 0.05. A *p*-value lower than 0.001 was considered to indicate a very high significance level. All analyses were performed using SAS software, version 9.4 (SAS Institute, Cary, North Carolina, CA, USA).

## 3. Results

Baseline characteristics are displayed in Table 1. Effect size among three different models was also examined (Table A1). It is also noted that the variance inflation value for the multicollinearity of the covariates included in this study ranged from 1.03 to 2.03, which means that the variables were independent. It is notable that the differences in the mean MMSE scores between depression change groups are considered highly statistically significant (*p* < 0.0001). Post hoc analysis using the Tukey method showed statistically significant differences between all groups (Table A2 and Table A3). Other covariates such as age, educational level, working status, participation in social activities, perceived health status, regular physical activities, and weight change of ≥5kg in the past year were also highly significant for both men and women (*p* < 0.0001). Household income was also significant for both sexes (*p* < 0.05).

Table 2 shows multiple regression analysis results between MMSE values and depression change groups. Group A was set as the reference value. For men, the regression coefficient is shown to have a decrease of 0.717 in Group B, a lesser decrease of 0.416 in Group C, and a greater decrease of 1.539 in Group D. Similarly, women’s cases in Group B show a regression coefficient of −0.629, a value of −0.430 in Group C, and a more negative regression coefficient of −1.413 in Group D. This trend of the degree of cognitive impairment being severe in the order of Group D, B, C, and A is also shown in Table A4, Table A5 and Table A6, which include parallel multiple regression analyses setting Group B–D as the reference, respectively.

It is notable that Group C shows the smallest decrease in the regression coefficient for both sexes; Group D, on the other hand, shows the largest decrease in the coefficient for both genders. The results of other covariates can also be observed: age groups for one show significant results. In particular, while the regression coefficient can be observed to increase until the age of 64, a definite decrease in the coefficient is seen in both men and women who are 75 years of age or older. Educational level is also a highly significant variable, as a lower educational level tends to result in a diminished regression coefficient in both sexes. Those with an educational level of elementary school or less, especially, show a strong decrease in the regression coefficient when compared with the group of university education or beyond. It is also notable that the regression coefficient for women is about twice that of men in each category, meaning a steeper difference trend from the reference value for women.

In terms of weight change, fluctuations and a decrease in weight were both seen to result in a decrease in the regression coefficient compared with no change, with fluctuations being especially significant. It is also noted that compared with those living with family over two generations, those living only with a spouse had the greatest regression coefficient, followed by those living with two generations. Women in rural areas show a lower regression coefficient, while region seems to have little significance in men. Low and mid-low household income levels show decreased regression coefficients in men, compared with the high household income level. In women, the mid-low household income group shows a greater regression coefficient compared with the high household income group, whereas the low household income group has a lesser one. Working status, participation in social activities, physical activities, alcohol intake, and perceived health status were revealed to be important factors, with those working, participating in social activities, doing regular physical activities, drinking, and with a more positive perception of their health status showing greater regression coefficient values. Smoking status and the number of chronic medical conditions seem to have little or no direct correlation with the MMSE score.

Table 3 shows the results of subgroup analysis of depression development with MMSE. In terms of age, a trend can be identified showing that groups of more advanced age have a smaller regression coefficient regardless of gender and depression development groups. Regarding working status, participation in social activities, and regular physical activities, those who responded positively (working, yes, and yes, respectively) show a bigger regression coefficient than those who responded negatively (non-working, no, and no) irrespective of gender and depression change groups. It is noteworthy that in both genders and all depression change groups, those without chronic medical conditions show a bigger regression coefficient than those who do suffer from such conditions.

## 4. Discussion

We investigated the association between the change in depressive state and cognitive function multi-directionally by comparing every case of depressive state change (normal to normal, normal to depressive, depressive to normal, depressive to depressive). Our findings reveal that those who have suffered from depression experience a decline in cognitive function. An interesting point is that people experience cognitive impairment not only in the case of depression-onset (normal to depressive) but also depression-remission (depressive to normal). Meanwhile, the latter case experiences moderate decline in cognitive function when compared with the former case. We also examined some meaningful results in terms of age, working status, participation in social activities, regular physical activities, and number of chronic medical conditions.

Our results suggest that those who experience a change in depressive state undergo cognitive impairment. In Table 2, the negative value of the regression coefficient for Group B indicates that the onset of depression is associated with a decline in cognitive abilities, which is consistent with the findings of numerous previous studies [4,5,20,21]. Meanwhile, the regression coefficient for Group C, also negative, indicates that remission of depression is associated with a decline in cognitive abilities as well. Numerous studies report the possibly persistent feature of cognitive impairment after remission of depression episodes [12,22,23,24]. Indeed, Maria Semkovska et al. state, “Deficits in selective attention, working memory, and long-term memory persist in remission from a major depressive episode and worsen with repeated episodes” [10]. These findings are in line with preceding studies suggesting that antidepressant medication only remedies patients’ mood disorders but cannot improve cognitive dysfunction [25,26]. It is inappropriate, however, to propose that antidepressant medication is useless, because there is evidence of the efficacy of drugs: “Antidepressant drugs mitigate cognitive dysfunction in some people with Major Depressive Disorder” [25]. Instead, we might be able to suggest that antidepressant medication cannot ‘fully’ improve cognitive dysfunction. Then, it is notable that those whose depressive symptoms remit experience less decline in cognitive abilities than those who develop depression. The negative value of the regression coefficient for Group B in Table A5, which sets Group C as the reference value, agrees with this finding.

There are also meaningful results which can be derived from Table 1 and Table 3. First, aging is associated considerably with decline in cognitive function, which is in line with common sense and preceding studies [27,28]. Not only do one’s cognitive abilities deteriorate as one gets older, but the degree of decline in cognitive impairment increases as well. This can be verified from our results: the older the population, the lower the mean MMSE score in Table 1 and the smaller the regression coefficient in Table 3. Second, those living a stimulating life experience less cognitive impairment accompanied with change in depressive state. Abovementioned ‘stimulating life’ corresponds to ‘working’, ‘participating in social activities’, and ‘doing regular physical activities’ in our study. In Table 3, the absolute values of regression coefficients of those who work, participate in social activities, and do regular physical activities are all smaller than the opposite. Numerous studies concerning this issue are consistent with the results [29,30,31,32,33,34]. Third, those with a greater number of chronic medical conditions experience more severe cognitive impairment accompanied with change in depressive state. This can be inferred from the following tendency: the greater the number of chronic medical conditions, the smaller the regression coefficient in Table 3. In this regard, Joshua Chodosh et al. suggest that depressive symptoms and chronic disease contribute to poorer cognitive function independently [35]. In other words, those with both depression and chronic disease experience more intense cognitive decline than those without the latter. These findings also support previous research suggesting that “performance on most cognitive measure was poorer in the presence of hypertension or DM as compared with other chronic disease” [36].

Some limitations should be noted. First, the possible bias due to the nature of the survey method should be addressed: the KLoSA database used for the study is based on self-reported investigation. Because it is not a clinical diagnosis but a subjective judgement by the individual, it may not represent the subject’s health condition as it is, whether intended or not. Second, the results may have been exaggerated for the reason that the passage of time during the investigation may accompany cognitive decline as mentioned above. Because our key variable—changes in depressive state—inevitably involves the passage of time, regression coefficients of Table 2 and Table 3 include not only the association between changes in depressive state and cognitive function but also cognitive decline accompanying aging. Third, the causal link between changes in depressive state and cognitive function cannot be addressed, because the study is not based on prospective study design. In this regard, it would be enlightening to investigate the causal relationship between them with a prospective study design.

The strength of our study, however, is that we analyzed cognitive abilities corresponding to depression change multi-directionally by comparing every case of depression change (normal to normal, normal to depressive, depressive to normal, and depressive to depressive), whereas numerous previous studies concentrated on cognitive impairment either when having developed depression or having been cured of depression. With this wider viewpoint, direct comparison between changes in cognitive abilities corresponding to two opposite depression change directions becomes possible, making the study more comprehensive. Another characteristic of the study is that it examines the degree of change in an individual’s own cognitive abilities, whereas most previous studies compared subjects’ cognitive abilities with those of other individuals. In other words, while most previous studies compared the cognitive abilities of a depressive with that of another who is in a normal mood, our study compared the degree of change in cognitive abilities between four depression change groups. Lastly, it is also noticeable that the MMSE, the measure of cognitive abilities, was employed as a raw index, not as a criterion for dividing normal cognition, mild cognitive impairment, and dementia. As the raw MMSE score is a continuous variable, it was possible to establish more meaningful intergroup comparisons and concrete results.

## 5. Conclusions

In summary, there is a significant association between changes in depressive state and cognitive function: regardless of the direction in which depressive state changes, cognitive function is damaged. The degree of cognitive function being impaired is greater in the case of depression-onset than that of depression-remission. It is also found that age, stimulating activities, and chronic conditions have a remarkable relevance for cognitive decline accompanied with change in depressive state. The strength of our research lies in the fact that interpretations were conducted by case-categorizing types of depression change, which makes the discussion more comprehensive. However, due to the limitations of the study design, causation cannot be addressed. Further studies using a prospective study design would be worthwhile.

## Figures and Tables

**Table 1:** Baseline characteristics of study population by MMSE (Mini-Mental State Examination).

Variables	Men					Women				
	Subjects		MMSE			Subjects		MMSE		
	*n*	%	Mean ± S.D		*p*-Value	*n*	%	Mean ± S.D		*p*-Value
**Changes in Depressive State**					<0.0001					<00.0001
Normal to Normal (Group A)	1386	45.7	25.947	2.662		1334	33.7	24.706	4.077	
Normal to Depressive (Group B)	476	15.7	24.487	3.802		566	14.3	23.339	4.896	
Depressive to Normal (Group C)	398	13.1	25.618	3.147		650	16.4	23.922	4.715	
Depressive to Depressive (Group D)	771	25.4	22.551	5.673		1408	35.57	21.031	5.932	
**Age**					<0.0001					<0.0001
45–54	582	19.2	26.440	2.296		821	20.7	26.055	2.464	
55–64	971	32.0	25.837	3.031		1159	29.3	25.144	3.180	
65–74	938	31.0	24.239	4.432		1144	28.9	22.543	4.534	
≥75	540	17.8	22.202	5.189		834	21.1	17.993	6.507	
**Education level**					<0.0001					<0.0001
Elementary school or less	950	31.3	22.878	5.160		2300	58.1	21.297	5.703	
Middle school	536	17.7	25.095	3.617		658	16.6	25.044	3.488	
High school	1046	34.5	25.815	2.904		833	21.1	25.772	3.117	
University or beyond	499	16.5	26.078	3.144		167	4.2	26.341	2.524	
**Region**					0.5899					0.0802
Metropolitan	1320	43.6	25.136	3.842		1767	44.6	23.508	5.055	
Rural	1711	56.5	24.560	4.307		2191	55.4	22.724	5.400	
**Working Status**					<0.0001					<0.0001
Working	1806	59.6	25.841	2.780		1248	31.5	24.901	3.488	
Non-working	1225	40.4	23.291	5.173		2710	68.5	22.233	5.710	
**Household income**					0.0148					0.0178
Low	746	24.6	22.724	5.224		1274	32.2	20.983	5.653	
Mid-low	762	25.1	24.790	3.720		932	23.6	23.523	4.685	
Mid-high	818	27.0	25.540	3.343		933	23.6	24.046	4.987	
High	705	23.3	26.194	3.022		819	20.7	24.711	4.484	
**Participation in social activities**					<.0001					<0.0001
No	680	22.4	22.500	5.852		1101	27.8	20.096	6.332	
Yes	2351	77.6	25.479	3.163		2857	72.2	24.222	4.264	
**Perceived health status**					<0.0001					<0.0001
Healthy	1618	53.4	25.936	2.723		1561	39.4	25.020	3.708	
Average	910	30.0	24.541	3.699		1363	34.4	23.045	4.907	
Unhealthy	503	16.6	21.678	6.306		1034	26.1	20.176	6.279	
**Regular physical activities**					<0.0001					<0.0001
Yes	1156	38.1	25.612	2.975		1213	30.7	24.788	3.699	
No	1875	61.9	24.317	4.622		2745	69.4	22.317	5.658	
**Smoking**					0.0972					0.4543
Current	1119	36.9	25.188	3.631		119	3.0	21.378	5.678	
Former	905	29.9	24.659	4.202		63	1.6	20.302	7.570	
Never	1007	33.2	24.528	4.512		3776	95.4	23.174	5.181	
**Alcohol intake**					0.1058					0.3768
Yes	1772	58.5	25.396	3.368		721	18.2	24.408	4.160	
No	1259	41.5	23.986	4.875		3237	81.8	22.777	5.434	
**Number of chronic medical conditions**					0.0366					0.1106
None	1345	44.4	25.687	3.122		1486	37.5	24.387	4.711	
1	898	29.6	24.793	3.851		1280	32.3	23.116	5.064	
≥2	788	26.0	23.335	5.322		1192	30.1	21.393	5.639	
**Weight change of ≥5kg in the past year**					<0.0001					<0.0001
No change	2282	75.3	25.309	3.491		2883	72.84	23.503	5.063	
Increase	101	3.3	24.228	4.025		214	5.41	23.888	4.111	
Decrease	369	12.2	22.751	5.803		454	11.47	21.211	5.749	
Fluctuations	279	9.2	23.667	5.055		407	10.28	21.686	5.890	
**Number of cohabiting generations**					0.2681					0.0028
Couple	1420	46.9	24.204	4.437		1896	47.9	22.760	4.998	
Two generations	1288	42.5	25.544	3.460		1502	38.0	24.003	5.110	
Over two generations	323	10.7	24.554	4.617		560	14.2	21.648	6.046	
**Total**	3031	100	24.811	4.121		3958	100	23.074	5.262	

**Table 2:** Results of analyzing factors associated with MMSE (Mini-Mental State Examination).

Variables	Men			Women		
	β	S.E	*p*-Value	β	S.E	*p*-Value
**Changes in Depressive State**						
Normal to Normal (Group A)	Ref.			Ref.		
Normal to Depressive (Group B)	−0.717	0.102	<0.0001	−0.629	0.097	<0.0001
Depressive to Normal (Group C)	−0.416	0.094	<0.0001	−0.430	0.087	<0.0001
Depressive to Depressive (Group D)	−1.539	0.108	<0.0001	−1.413	0.104	<0.0001
**Age**						
45–54	Ref.			Ref.		
55–64	0.362	0.089	<0.0001	0.423	0.079	<0.0001
65–74	0.123	0.125	0.3240	−0.460	0.122	0.0002
≥75	−0.722	0.174	<0.0001	−2.836	0.167	<0.0001
**Education level**						
Elementary school or less	−2.423	0.169	<0.0001	−4.521	0.179	<0.0001
Middle school	−0.602	0.160	0.0002	−1.243	0.175	<0.0001
High school	−0.232	0.117	0.0464	−0.434	0.150	0.0039
University or beyond	Ref.			Ref.		
**Region**						
Metropolitan	Ref.			Ref.		
Rural	−0.065	0.102	0.5243	−0.332	0.106	0.0018
**Working Status**						
Working	Ref.			Ref.		
Non–working	−0.698	0.091	<0.0001	−0.646	0.080	<0.0001
**Household income**						
Low	−1.150	0.145	<0.0001	−0.295	0.141	0.0363
Mid–low	−0.145	0.094	0.0060	0.267	0.119	0.0251
Mid–high	−0.145	0.094	0.1205	0.137	0.104	0.1889
High	Ref.			Ref.		
**Participation in social activities**						
No	−1.734	0.127	<0.0001	−1.654	0.105	<0.0001
Yes	Ref.			Ref.		
**Perceived health status**						
Healthy	Ref.			Ref.		
Average	−0.343	0.068	<0.0001	−0.442	0.073	<0.0001
Unhealthy	−2.281	0.145	<0.0001	−2.085	0.116	<0.0001
**Regular physical activities**						
Yes	0.587	0.077	<0.0001	0.671	0.072	<0.0001
No	Ref.			Ref.		
**Smoking**						
Current	0.371	0.121	0.0022	0.109	0.333	0.7445
Former	0.187	0.126	0.1384	−0.649	0.399	0.1040
Never	Ref.			Ref.		
**Alcohol intake**						
Yes	0.357	0.093	0.0001	0.541	0.108	<0.0001
No	Ref.			Ref.		
**Number of chronic medical conditions**						
None	Ref.			Ref.		
1	0.077	0.100	0.4414	0.261	0.115	0.0226
≥2	−0.148	0.126	0.2413	0.108	0.142	0.4461
**Weight change of ≥5kg in the past year**						
No change	Ref.			Ref.		
Increase	−0.172	0.128	0.1785	0.086	0.105	0.4153
Decrease	−0.677	0.123	<0.0001	−0.395	0.109	0.0003
Fluctuations	–1.115	0.189	<0.0001	−1.395	0.175	<0.0001
**Number of cohabiting generations**						
Couple	0.702	0.171	<0.0001	0.996	0.156	<0.0001
Two generations	0.308	0.166	0.0626	0.423	0.157	0.0070
Over two generations	Ref.			Ref.		

**Table 3:** Results of subgroup analysis of depression development with MMSE.

Variables			Changes in Depressive State									
			Normal to Normal(Group A)	Normal to Depressive(Group B)			Depressive to Normal(Group C)			Depressive to Depressive(Group D)		
			β	β	S.E	*p*-Value	β	S.E	*p*-Value	β	S.E	*p*-Value
**Men**	**Age**	45–54	Ref.	−0.6418	0.2217	<0.0001	0.08	0.1806	0.6576	−0.6064	0.2442	0.013
		55–64	Ref.	−0.5754	0.1482	0.0001	−0.1251	0.1306	0.3379	−1.1959	0.1528	<0.0001
		65–74	Ref.	−0.9235	0.1996	<0.0001	−0.5082	0.1655	0.0021	−1.5875	0.1814	<0.0001
		≥75	Ref.	−1.0562	0.2416	<0.0001	−0.8721	0.2569	0.0007	−2.2595	0.2417	<0.0001
	**Working status**	Working	Ref.	−0.6906	0.1172	<0.0001	−0.1564	0.0976	0.109	−1.0477	0.1103	<0.0001
		Non–working	Ref.	−0.7559	0.1727	<0.0001	−0.6893	0.1796	0.0001	−1.8139	0.1787	<0.0001
	**Participation in social activities**	No	Ref.	−1.0705	0.3051	0.0005	−1.1854	0.2981	<.0001	−2.4268	0.2821	<0.0001
		Yes	Ref.	−0.6741	0.1067	<0.0001	−0.214	0.0923	0.0204	−1.1992	0.105	<0.0001
	**Regular physical activities**	Yes	Ref.	−0.5601	0.1361	<0.0001	−0.3747	0.134	0.0052	−1.425	0.1509	<0.0001
		No	Ref.	−0.8295	0.1499	<0.0001	−0.3982	0.1294	0.0021	−1.6323	0.1385	<0.0001
	**Number of chronic medical conditions**	None	Ref.	−0.5652	0.1382	<0.0001	−0.0218	0.1262	0.8631	−1.1953	0.1523	<0.0001
		1	Ref.	−0.8462	0.2021	<0.0001	0.0526	0.1637	0.748	−1.2027	0.1902	<0.0001
		≥2	Ref.	−0.8291	0.2142	<0.0001	−1.2408	0.199	0.0001	−2.166	0.2096	<0.0001
**Women**	**Age**	45–54	Ref.	−0.1882	0.1775	<0.0001	−0.1052	0.1711	0.5388	−0.5266	0.1991	0.0082
		55–64	Ref.	−0.7177	0.1298	<0.0001	−0.2076	0.108	0.0547	−1.2455	0.1336	<0.0001
		65–74	Ref.	−0.6797	0.1783	0.0001	−0.3658	0.1523	0.0163	−1.6416	0.1714	<0.0001
		≥75	Ref.	−0.74	0.2771	0.0076	−0.9771	0.2568	0.0001	−1.7301	0.2672	<0.0001
	**Working status**	Working	Ref.	−0.3845	0.1479	0.0093	−0.3425	0.1318	0.0094	−1.0245	0.1524	<0.0001
		Non–working	Ref.	−0.7139	0.1253	<0.0001	−0.5034	0.1124	<.0001	−1.5261	0.1302	<0.0001
	**Participation in social activities**	No	Ref.	−1.198	0.257	<0.0001	−1.1648	0.2368	<.0001	−2.1144	0.2333	<0.0001
		Yes	Ref.	−0.5096	0.1038	<0.0001	−0.2597	0.0918	0.0047	−1.2726	0.1091	<0.0001
	**Regular physical activities**	Yes	Ref.	−0.4491	0.1389	0.0012	−0.3678	0.1263	0.0036	−1.502	0.1478	<0.0001
		No	Ref.	−0.6907	0.1316	<0.0001	−0.5256	0.1181	<.0001	−1.425	0.1301	<0.0001
	**Number of chronic medical conditions**	None	Ref.	−0.3997	0.1466	0.0064	−0.2438	0.1379	0.077	−1.4042	0.1734	<0.0001
		1	Ref.	−0.6238	0.18	0.0005	−0.1692	0.1541	0.272	−1.1589	0.1664	<0.0001
		≥2	Ref.	−1.0232	0.1908	<0.0001	−0.9455	0.1702	<.0001	−1.9229	0.1859	<0.0001

## Appendix A

**Table A1:** The effect size of three different models.

Variables	Crude Model			Model 1			Model 2		
	*β*	*S.E.*	*p*-Value	*β*	*S.E.*	*p*-Value	*β*	*S.E.*	*p*-Value
***Male***									
Normal to Normal	Ref.			Ref.			Ref.		
Normal to Depressive	−1.408	0.112	<0.0001	−1.071	0.106	<0.0001	−0.717	0.102	<0.0001
Depressive to Normal	−0.911	0.102	<0.0001	−0.563	0.097	<0.0001	−0.416	0.094	<0.0001
Depressive to Depressive	−2.691	0.134	<0.0001	−2.087	0.120	<0.0001	−1.539	0.108	<0.0001
***Female***									
Normal to Normal	Ref.			Ref.			Ref.		
Normal to Depressive	−1.207	0.101	<0.0001	−0.934	0.098	<0.0001	−0.629	0.097	<0.0001
Depressive to Normal	−0.871	0.092	<0.0001	−0.547	0.088	<0.0001	−0.430	0.087	<0.0001
Depressive to Depressive	−2.476	0.114	<0.0001	−1.873	0.106	<0.0001	−1.413	0.104	<0.0001

Model 1 adjusted age, educational level, region, household income, working status. Model 2 adjusted age, educational level, region, household income, working status, participants in social activities, perceived health status, regular physical activities, smoking, alcohol intake, number of chronic medical conditions, weight change of ≥5kg in the past year, number of cohabiting generations.

**Table A2:** Post hoc analysis (Tukey method) of changes in depressive state for male individuals.

Comparisons Significant at the 0.05 Level are Indicated by ***.				
Interesting Group Comparison	Difference Between Means	Simultaneous 95% Confidence Limits		
0–2	1.03292	0.72017	1.34568	***
0–1	1.77209	1.45383	2.09036	***
0–3	3.71874	3.46277	3.97471	***
2–0	−1.03292	−1.34568	−0.72017	***
2–1	0.73917	0.3447	1.13364	***
2–3	2.68582	2.33964	3.03199	***
1–0	−1.77209	−2.09036	−1.45383	***
1–2	−0.73917	−1.13364	−0.3447	***
1–3	1.94665	1.59548	2.29781	***
3–0	−3.71874	−3.97471	−3.46277	***
3–2	−2.68582	−3.03199	−2.33964	***
3–1	−1.94665	−2.29781	−1.59548	***

0: Normal to Normal, 1: Normal to Depressive, 2: Depressive to Normal, 4: Depressive to depressive.

**Table A3:** Post hoc analysis (Tukey method) of changes in depressive state for female individuals.

Comparisons Significant at the 0.05 Level are Indicated by ***.				
Interesting Group Comparison	Difference Between Means	Simultaneous 95% Confidence Limits		
0–2	1.5348	1.20371	1.86589	***
0–1	1.98415	1.6327	2.3356	***
0–3	4.59168	4.3253	4.85806	***
2–0	−1.5348	−1.86589	−1.20371	***
2–1	0.44935	0.03806	0.86064	***
2–3	3.05688	2.71541	3.39836	***
1–0	−1.98415	−2.3356	−1.6327	***
1–2	−0.44935	−0.86064	−0.03806	***
1–3	2.60753	2.24629	2.96878	***
3–0	−4.59168	−4.85806	−4.3253	***
3–2	−3.05688	−3.39836	−2.71541	***
3–1	−2.60753	−2.96878	−2.24629	***

0: Normal to Normal, 1: Normal to Depressive, 2: Depressive to Normal, 4: Depressive to depressive.

**Table A4:** Results of analyzing factors associated with MMSE (Group B as a reference).

Variables	Men			Women		
	β	S.E	*p*-Value	β	S.E	*p*-Value
**Changes in Depressive State**						
Normal to Normal	0.7169	0.1023	<0.0001	0.6289	0.0967	<0.0001
Normal to Depressive	Ref.			Ref.		
Depressive to Normal	0.3009	0.1072	0.005	0.1988	0.0976	0.0417
Depressive to Depressive	−0.8216	0.1249	<0.0001	−0.7841	0.1097	<0.0001

All covariates are adjusted for the analysis.

**Table A5:** Results of analyzing factors associated with MMSE (Group C as a reference).

Variables	Men			Women		
	β	S.E	*p*-Value	β	S.E	*p*-Value
**Changes in Depressive State**						
Normal to Normal	0.4161	0.0944	<0.0001	0.4301	0.0865	<0.0001
Normal to Depressive	−0.3009	0.1072	0.005	−0.1988	0.0976	0.0417
Depressive to Normal	Ref.			Ref.		
Depressive to Depressive	−1.1224	0.1175	<0.0001	−0.9829	0.1026	<0.0001

All covariates are adjusted for the analysis.

**Table A6:** Results of analyzing factors associated with MMSE (Group D as a reference).

Variables	Men			Women		
	β	S.E	*p*-Value	β	S.E	*p*-Value
**Changes in Depressive State**						
Normal to Normal	1.5385	0.1083	<0.0001	1.413	0.104	<0.0001
Normal to Depressive	0.8216	0.1249	<0.0001	0.7841	0.1097	<0.0001
Depressive to Normal	1.1224	0.1175	<0.0001	0.9829	0.1026	<0.0001
Depressive to Depressive	Ref.			Ref.		

All covariates are adjusted for the analysis.
